# Risk factors of peripheral venous catheter-related complication and infection in children with bronchopneumonia

**DOI:** 10.1186/s12879-023-08540-1

**Published:** 2023-09-15

**Authors:** Hong-mei Li, Li-li Wan, Cai-xiang Jin, Guo-ying Zhang, Hui Yang, Xiao-yu Zhang

**Affiliations:** 1 Department of General Services, Nanjing Hospital of Integrated Traditional Chinese and Western Medicine, No. 179 of Xiaolingwei Street, Xuanwu District, Nanjing, 210014 China; 2 Department of Paediatrics, Nanjing Hospital of Integrated Traditional Chinese and Western Medicine, Nanjing, 210014 China; 3 Department of Infection Management, Nanjing Hospital of Integrated Traditional Chinese and Western Medicine, Nanjing, 210014 China; 4 Department of Clinical laboratory, Nanjing University of Traditional Chinese Medicine, Nanjing, 210014 China; 5 Department of Paediatrics, Nanjing University of Traditional Chinese Medicine, Nanjing, 210014 China

**Keywords:** Children, Peripheral venous catheter, Retention time, Indwelling part, Infection factors

## Abstract

**Objective:**

To investigate the risk factors associated with the peripheral venous catheter-related complication and infection in children with bronchopneumonia.

**Methods:**

A total of 185 patients were divided into case group (n = 114) and control group (n = 71) according to the presence of catheter-related infection and complications related to indwelling needle. We performed a multivariate logistic regression analysis to explore the risk factors associated with the infection.

**Results:**

Age was divided into 4 categories (0 < age ≤ 1, 1 < age ≤ 3, 3 < age ≤ 6, age > 6). The case group had a higher percentage of patients with 0 < age ≤ 1 than the control group (21% vs. 9.7%) and the age distribution was significant different between the two groups (*P* = 0.045). The case group had a longer retention time than the control group (≥ 3 days: 56% vs. 35%, *P* < 0.001). The results of binary logistics regression analysis revealed that the indwelling time and indwelling site were the factors that influenced the complications or bacterial infection. Among the three indwelling sites, the hand is more prone to infection and indwelling needle-related complications than the head (OR: 2.541, 95% CI 1.032 to 6.254, P = 0.042). The longer the indwelling time, the more likely the infection and indwelling needle related complications (OR: 2.646, 95% CI 1.759 to 3.979, P< 0.001).

**Conclusion:**

Indwelling time and indwelling site are the influencing factors of complications or bacterial infection, which should be paid more attention to prevent the catheter-related infection in children with bronchophenumonia.

## Background

Peripheral intravenous catheterisation is the most common invasive operation in hospitalised patients, with 70% of hospitalised patients receiving peripheral intravenous catheterisation and intravenous drug therapy [1]. Complications associated with intravenous indwelling catheters include peripheral phlebitis, which is the most common complication with an incidence of 2.3–60%, followed by venous extravasation and catheter-associated bacteraemia [2]. The pathogenesis of catheter-related infection may include: (1) bacteria on the skin surface colonise the catheter surface and tip during or after puncture; and (2) microorganisms from other foci of infection disseminate in the bloodstream and bacteria colonise the lumen [3]. Most relevant studies have focused on the retention time of indwelling needles, followed by catheter needle-related infections, with fewer studies conducted on bacterial microbiology and even fewer on paediatric patients with a certain specificity of peripheral vasculature. Therefore, it is essential to identify the risk for catheter-related infection at an early stage, especially during the pandemic of COVID-19 [4–6].

For this reason, the present study aimed to explore the risk factors associated with the peripheral catheter-related complication and infection in children with bronchopneumonia. This will provide information for further studies on indwelling needle-associated infections in paediatric patients, identify correlations between peripheral vein placement-related complications and puncture sites and catheter indwelling times in paediatric patients, and provide evidence for preventing peripheral vein catheter-related complications [7, 8].

## Methods

### Study design and population

This study was a retrospective, single-center, case-control study. We involved 185 hospitalised paediatric patients (aged 0–12 years) who were diagnosed with bronchopneumonia between January 2019 and December 2020 in our hospital. They were divided into a case group and control group according to the occurrence of catheter-related infection and complications related to indwelling needles. There were 114 cases in the control group and 71 in the case group. This study was approved by the ethics committee of our hospital and the requirement of informed consents was waived due to the retrospective design.

For all patients, inclusion criteria were as follows: (1) Patients who had received an intravenous indwelling needle for infusion; (2) patients with a consistent admission diagnosis; (3) patients with a consistent therapeutic drug regimen; and (4) the informed consent and cooperation of the child and family. Exclusion criteria were as follows: (1) Patients with severe diseases of the liver, kidney, urinary system, respiratory tract, circulatory system, brain and haematopoietic system; and (2) patients whose indwelling needles had been removed midway through the treatment or whose treatment had been terminated for other unexpected reasons.

### Sampling and testing methods

#### Skin sampling method at the puncture site

Samples were taken from the skin around the puncture site of each patient once before venipuncture and once before needle removal after sterilisation. The local infection caused by the indwelling needle was judged by observing whether the puncture site appeared red, swollen, painful, purulent or demonstrated signs of other inflammatory reactions. A sterile cotton swab soaked with the corresponding neutralising agent together with a 5 cm $\times$ 5 cm specification plate was evenly rubbed 5 times horizontally and vertically back and forth around the puncture site of the indwelling needle, and then the cotton swab was rotated and placed in 10 mL of the corresponding neutralising agent after cutting off the part of the swab that had been in contact with the skin using sterilised scissors. Subsequently, l ml of the sample was extracted for testing.

#### Needle sampling method for indwelling needles

The local area was first disinfected, and the indwelling needle catheter was then removed aseptically. Subsequently, 2–3 cm of the proximal end was cut off with sterilised scissors, and the needle was placed in the corresponding eluent aseptically, with a l-mL sample taken for testing. The type of catheter and puncture site were noted.

#### Storage and transportation of specimens

The specimens were collected by members of the study group in strict accordance with the standardised criteria, and after standard collection, they were placed in a special specimen box and refrigerated at 2℃–8℃.

#### Test method

Each specimen was inoculated with either an aerobic or fungal medium and incubated at 35℃. The results were observed after day 2 (the fungal culture was placed in an incubator at 25℃). Blank and positive controls were set to exclude the interference of swabs, reagents, nutrient solution and other factors on the experiment, and provide a comprehensive analysis.

### Observation index

Indicators for the withdrawal of the peripheral intravenous indwelling needles, using the withdrawal criteria for vascular access devices in the 2016 edition of the *Standards of Practice for Infusion Therapy* [4, 9], are as follows: the withdrawal of peripheral intravenous catheters, whether in or not in an infusion, is required whenever clinically indicated based on the results of a puncture site assessment and/or clinical signs and symptoms of systemic complications (such as bloodstream infection).

The signs and symptoms of complications or infection mainly include: (1) any degree of pain and/or tenderness or pain without palpation; (2) colour change (erythema or whitening); (3) change in skin temperature (hot or cold); (4) oedema; (5) sclerosis; (6) fluid or pus exuding from the puncture site; and (7) other types of dysfunction (e.g. resistance to flushing and no blood return). The patients with the above indications for extubation were regarded as the case group, and the patients without the above indications for extubation at the same position and time were regarded as the control group.

When the indwelling needle was removed, the puncture site conditions were recorded: phlebitis (0–4), extravasation (0–4), pain (yes, no) and other types of dysfunction (yes, no). The criteria for determination were the phlebitis scale from the 2016 edition of the *Standards of Practice for Infusion Therapy* and the 2009 edition of the Infusion Nurses Society standard scale for grading exudate.

### Quality control

(1) Patients were selected according to the inclusion criteria, and the procedures and requirements were explained to the children and their families before the operation to obtain their cooperation.

(2) All operations were performed by professionals according to standard operating procedures.

(3) Informed consent was given, and attention was paid to the protection of the puncture area during the use of the peripheral intravenous indwelling needles; the puncture area was kept clean and dry.

(4) Sampling specimens were sent to the laboratory for testing within 2 h.

### Statistical methods

SAS 9.4 software (**SAS** Institute Inc, Cary, NC) was used for all data analysis. Frequency and percentage were used to describe each categorical variable, and the χ^2^ test was applied to compare the differences in the distribution of each factor between the case and control groups. Logistic multivariate regression analysis was used for identifying the independent factors associated with the catheter-related complication and infection, and a two-tailed *P value* < 0.05 indicated that the differences were statistically significant.

## Results

### Age comparison between the two groups

After reclassifying for age, a frequency table was developed and χ^2^ test conducted. Age was divided into ‘1 year or younger’ (0 < age ≤ 1), ‘1–3 years’ (1 < age ≤ 3), ‘3–6 years’ (3 < age ≤ 6) and ‘6 years or older’ (age > 6) according to the segmentation standard and data distribution.

The difference in age distribution between the case group and control group (χ^2^ = 8.185, *P* = 0.045) was statistically significant (Table 1).

**Table 1:** Comparison of age, retention time, indwelling parts between the two groups

Variables	Control group (N = 114)	Case group (N = 71)	χ^2^	*P*
Age, years			8.185	0.045
0–1	11 (9.7%)	15 (21%)		
1–3	42 (37%)	23 (32%)		
3–6	51(45%)	22 (31%)		
> 6	10 (8.8%)	11 (15%)		
Retention time			27.015	< 0.001
1 day	57 (50%)	9 (13%)		
2 days	17 (15%)	22 (31%)		
≥ 3 days	40 (35%)	40 (56%)		
Indwelling parts			6.290	0.045
Head	17 (15%)	21 (30%)		
Hand	50 (44%)	29 (41%)		
Feet	47 (41%)	21 (30%)		

Data are expressed as counts (percentage) and compared using the chi-square test

### Comparative analysis of the retention time of the two groups

After the retention time of the two groups had been reclassified, a frequency table was developed and χ^2^ test conducted. The retention time was divided into three subgroups: ‘1 day’, ‘2 days’ and ‘3 days or more’. The indwelling time of the venous indwelling needle in the control group was generally 1 day (50%), whereas the indwelling time in the case group was 3 days or more (56%). The complications and bacterial colonisation in the case group generally lasted 3 days or more (56%). The difference in the distribution of retention time between case group and control group (χ^2^ = 27.015, *P* < 0.001) was statistically significant (Table 1).

### Comparative analysis of indwelling sites between the case and control groups

The indwelling sites of the two groups were reclassified, dividing them into three subgroups: head, hand and foot. The hand is the most common application site for both group (control group: 44%, case group: 41%). To explore the differences in indwelling needle sites, a χ^2^ test was then performed. And the difference in the distribution of indwelling sites in the two groups (χ^2^ = 6.290, *P* = 0.045) was statistically significant (Table 1).

### Risk factor analysis

A binary logistic regression model was adopted, and a stepwise regression method was used for modelling. The entry level of variables was set as 0.10, and the elimination level was set as 0.05. The analysis was performed with infection and indwelling needle-related complications as dependent variables, age, indwelling time and indwelling site as independent variables. The indwelling part sets the dummy variable with the head as the reference. The retention time enters the equation as a continuous variable. The results revealed that the indwelling time and indwelling site were the factors that influenced the complications or bacterial infection. Among the three indwelling sites, the hand is more prone to infection and indwelling needle-related complications than the head. The longer the indwelling time, the more likely the infection and indwelling needle related complications. As shown in Table 2, compared with those children with an indwelling site of head, those with an indwelling site of hands (OR: 2.541, 95% CI 1.032 to 6.254, *P* = 0.042) had a higher risk to have complications or bacterial infection.

**Table 2:** Results of multivariate logistic regression analysis

Parameters	Odds ratio	95% confidence interval	estimate(β)	Standard error	Wald X^2^	*P*
Intercept			-2.552	0.487	27.517	< 0.001
Position(hand vs. head)	2.541	1.032 to 6.254	0.933	0.460	4.118	0.042
Position(foot vs. head)	0.583	0.261 to 1.304	-0.539	0.411	1.724	0.189
Time	2.646	1.759 to 3.979	0.973	0.208	21.828	< 0.001

## Discussion

Understanding risk factors is a crucial first step in lowering CRI-related morbidity and death. In the present study, we explored the risk factors associated with the peripheral venous catheter-related complications and infection in children with bronchopneumonia and examined whether the indwelling site and retention time had an impact on the bacterial colonization and catheter-related infection. The main findings can be summarized as follows: (1) compared with the control group, the case group were more likely to have a lower age, a longer retention time of the indwelling needle; (2) catheter-related complications or bacterial infection were more likely to occur in those children with an indwelling site of hand; (3) after adjusting other confounding factors, a longer retention time and an indwelling site of hand were independent risk factors of catheter-related complications and infection.

The *Guidelines for the Prevention of Endovascular Catheter-Associated Infections*, jointly published by the American Critical Care Association, the Infectious Diseases Society and others, recommend that adult peripheral venous catheters be replaced every 72 to 96 h to reduce the risk of phlebitis and catheter-associated infections. However, the guidelines do not recommend routine replacement for paediatric patients and high-risk categories of catheters. Domestic and international studies on adults have demonstrated that catheter-associated infections are related to the puncture site. Moreover, Hu et al. revealed [10] that disposing of a tube in the elbow fossa increases the risk of mechanical phlebitis, with a risk 4.109 times greater than that of the dorsal hand placement site, and that caregivers should avoid leaving a catheter in this area. The results of the present study demonstrate that the possibility of complications and bacterial colonisation at the hand site is greater. Relevant studies have focused more frequently on the retention duration of peripheral venous indwelling needles [11, 12], followed by studies on needle-associated infections [13–16], but fewer studies have been conducted on the site of indwelling needles [17] and even fewer on the site of indwelling needles in relation to bacteria; studies on paediatric patients with certain special peripheral vasculature characteristics are rare and rarely reported in China.

From contradictory studies in children, it is still unclear if the position of the catheter’s tip and catheter-related complications are related. Catheters inserted in non-central veins were reported to give secure and dependable intravenous access in several pediatric studies [18, 19], while others revealed that catheter terminating in non-central veins carry a greater risk of complications [20, 21]. Because central veins are defined differently in different research, it is difficult to compare these findings. Given the size of our cohort, this is the first study to investigate the risk factors associated with catheter-related infection and complication in children with bronchopneumonia. In this study, we used a nested case–control study to determine that both the duration of peripheral venous placement and the site of puncture are factors influencing peripheral venous catheter-associated infections and needle-associated complications in children. To avoid complications in peripheral intravenous placement, the following nursing recommendations are proposed: follow catheter maintenance procedures strictly; apply moist heat to the placement limb before placement; puncture gently to reduce damage to the vessels and drug stimulation; ensure that the catheter is unobstructed when fixed; measure the patient’s arm circumference daily to ensure the catheter is unobstructed; according to the patient’s personality, ensure that they have a good awareness of the importance of catheter maintenance and answer any concerns in a timely manner; distribute brochures before discharge, explain the precautions and daily care and record all information in detail. Additionally, patients and their family members should be regularly trained in catheterisation care to make them aware of the importance of peripheral venous catheterisation; teach patients to maintain the catheter and the daily protection methods; instruct them not to immerse the puncture site in water, not to do strenuous exercise and to inform medical staff immediately if they experience any redness, swelling or pain; nurse–patient communication should be strengthened and a good nurse–patient relationship established; and targeted psychological counselling should be arranged in a timely manner and according to the patients’ condition and mental health. In terms of mental health, patients should be informed of previous success stories to improve their confidence in overcoming the disease and improve their treatment compliance and cooperation [22–25].

However, this paper did not analyse factors such as the materials used in the manufacture of peripheral venous catheters or the number of punctures children receive because of the difficulty in sampling specimens, the long treatment duration and the limited number of samples. These are among the limitations of this study. Our next study will involve a multicentre study with a large sample size to further justify our results.

## Conclusion

This study indicated the key role of indwelling retention time and site in the pathogenesis of catheter-related complications in children with respiratory tract diseases. Clinical application should be based on the actual situation and timely analysis of factors such as the length of placement and the puncture site in children, and effective measures should be taken to prevent and control the occurrence of local infection related to peripheral venous catheters and reduce the risk of infection to ensure patient treatment outcomes.

## Data Availability

The datasets used and analyzed during the current study are available from the corresponding author on reasonable request.
